# Supplementary material for: Metabolomics of Solanum lycopersicum Infected with Phytophthora infestans Leads to Early Detection of Late Blight in Asymptomatic Plants
Source: Molecules. 2018 Dec 15;23(12):3330. doi: 10.3390/molecules23123330 (PMC6320815; doi:10.3390/molecules23123330)
Supplement: Supplementary file 1 [file molecules-23-03330-s001.zip › molecules-387846-SI/ESM_Figures_.doc]

***Electronic Supplementary Material***

Metabolomics of *Solanum lycopersicum* infected with *Phytophthora infestans* leads to early detection of late blight in asymptomatic plants

**Paula Galeano^1,2,3^, Fábio Neves dos Santos^3^,** **Samantha Zanotta^4^, Marcos Nogueira Eberlin^3^, Chiara Carazzone^1^,***

^1^ Laboratory of Advanced Analytical Techniques in Natural Products. Universidad de los Andes, Bogotá 111711, Colombia; [plgaleanog@gmail.com](mailto:plgaleanog@gmail.com) (P.G); [c.carazzone@uniandes.edu.co](mailto:c.carazzone@uniandes.edu.co) (C.C).

^2^ Bioprospección de los Productos Naturales Amazónicos, Facultad de Ciencias Básicas, Universidad de la Amazonia, Florencia [180002](https://codigo-postal.co/colombia/cp/180002/), Colombia.

^3^ ThoMSon Mass Spectrometry Laboratory, University of Campinas, Institute of Chemistry, Campinas 13083-970, Brazil; [fabiof6@gmail.com](mailto:fabiof6@gmail.com) (F.N); [mneberlin@gmail.com](mailto:mneberlin@gmail.com) (M.E).

^4^ Laboratório de Diagnostico Fitopatológico, Instituto Biológico, São Paulo, São Paulo, 04014-900, Brazil; [sa_zanotta@terra.com.br](mailto:sa_zanotta@terra.com.br) (S.Z).

* Correspondence: [c.carazzone@uniandes.edu.co](mailto:c.carazzone@uniandes.edu.co); Tel.: +57 1 3394949 ext 1480.

**Figure S1.** (**a**) LC-ESI (+)-MS Chromatographic profiles and (**b**) Cloud Plot of metabolites detected in infected tomato samples (4, 12, 24, 36, 48, 60, 72 and 96 hpi) and the control. The red circles correspond to the statistical significance of each molecular feature given by the p-value (0.01). Bonferroni correction was used to adjust p-value to (0.01/45 = 0.0002).

**Figure S2.** (**a**) LC-ESI (-)-MS chromatographic profiles and (**b**) Cloud Plot of metabolites detected in infected tomato samples (4, 12, 24, 36, 48, 60, 72 and 96 hpi) and the control. The red circles correspond to the statistical significance of each molecular feature given by the p-value (0.01). Bonferroni correction was used to adjust p-value to (0.01/45 = 0.0002).

**Figure S3.** Principal component analysis (PCA) score plot of infected tomato samples (4, 12, 24, 36, 48, 60, 72 and 96 hpi), quality control (QC) and the control (C) analyzed by LC-ESI (+)-MS. The coding ***4-hpi*** means the analysis in positive ionization mode of the samples collected at four hours post infection. Coding ***C*** means the analysis in positive ionization mode of the controls. The QC samples consisted of a pool of all the different inoculation times and controls and were analyzed at the beginning and at the end of each batch and after every 10 injections.

**Figure S4.** Principal component analysis (PCA) score plot of infected tomato samples (4, 12, 24, 36, 48, 60, 72 and 96 hpi), and the negative control (C_neg) analyzed by LC-ESI (-)-MS. The coding ***4-hpi_neg*** means the analysis in negative ionization mode of the samples collected at four hours post infection. Coding ***C_neg*** means the analysis in negative ionization mode of the controls.

**Figure S5.** Heat map plot of the relative abundance of metabolites in infected tomato samples (4, 12, 24, 36, 48, 60, 72 and 96 hpi) and the control analyzed by LC-ESI (+)-MS. The parameters used were: Pearson distance; complete clustering algorithm and test ANOVA. Coding ***4-hpi*** means the analysis in positive ionization mode of the samples collected at four hours post infection. Coding ***C*** means the analysis in positive ionization mode of the controls.

**Figure S6.** Heat map plot of the relative abundance of metabolites in infected tomato samples (4, 12, 24, 36, 48, 60, 72 and 96 hpi) and the control analyzed by LC-ESI (-)-MS. The parameters used were: Pearson distance; complete clustering algorithm and test ANOVA. The coding ***4-hpi_neg*** means the analysis in negative ionization mode of the samples collected at four hours post infection. Coding ***C_neg*** means the analysis in negative ionization mode of the controls.

| **a**    **c**   | **b**    **d**   |
| --- | --- |

**Figure S7.** PLS-DA scores plot of infected tomato samples (4, 12, 24 and 36 hpi) analyzed by (**a)** LC-ESI (+)-MS (**b)** LC-ESI (-)-MS. OPLS-DA S-plots of the discriminant ions of the cluster I (early infection) obtained by (**c)** LC-ESI (+)-MS (**d)** LC-ESI (-)-MS. Coding ***4-hpi*** means the analysis in positive ionization mode of the samples collected at four hours post infection. Coding ***4-hpi_neg*** means the analysis in negative ionization mode of the samples collected at four hours post infection.

| **a**    **c**   | **b**    **d**   |
| --- | --- |

**Figure S8.** PLS-DA scores plot of infected tomato samples (48, 60, 72 and 96 hpi) analyzed by (**a)** LC-ESI (+)-MS (**b)** LC-ESI (-)-MS. OPLS-DA S-plots of the discriminant ions of the cluster II (late infection) obtained by (**c)** LC-ESI (+)-MS (**d)** LC-ESI (-)-MS. Coding ***48-hpi*** means the analysis in positive ionization mode of the samples collected at forty-eight hours post infection. Coding ***48-hpi_neg*** means the analysis in negative ionization mode of the samples collected at forty-eight hours post infection.

| **a**   | |
| --- | --- |
| **b**   | **c**   |
| **d**   | **e**   |

**Figure S9.** (**a).** OPLS-DA permutation test from LC-ESI (+)-MS data (4-96 hpi and control). Empirical p-values R2Y: p < 0.01 (0/100) and Q2: p < 0.01 (0/100). PLS-DA method validation of infected sample classification from LC-ESI (+)-MS data (4-96 hpi and control). Permutation tests based on prediction accuracy during training (**b**) and separation distance (**c**). Cross validation (CV) test based on leave-one-out –LOO (**d**), and 10-fold (**e**) using different number of components. The red circle indicates the best classifier. The p value based on permutation is p < 5e-04 (0/2000).

| **a**   | |
| --- | --- |
| **b**   | **c**   |
| **d**   | **e**   |

**Figure S10.** (**a).** OPLS-DA permutation tests from LC-ESI (-)-MS data (4-96 hpi and control). Empirical p-values R2Y: p < 0.01 (0/100) and Q2: p < 0.01 (0/100). PLS-DA permutation test from LC-ESI (-)-MS data (4-96 hpi and control). Permutation tests based on prediction accuracy during training (**b**) and separation distance (**c**). Cross validation (CV) test based on leave-one-out –LOO (**d**), and 10-fold (**e**) using different number of components. The red circle indicates the best classifier. The p value based on permutation is p < 5e-04 (0/2000).

**a**

**b**

**c**

**Figure S11.** MALDI (+)-MS profiles of tomato plants infected by *P. infestans* using MBT matrix: (**a**) Control; (**b**) early infection - 12 hpi and (**c**) late infection - 96 hpi.

**a**

**b**

**c**

**Figure S12.** MALDI (-)-MS profiles of tomato plants infected by *P. infestans* using DHB matrix: (**a**) Control; (**b**) early infection - 12 hpi and (**c**) late infection - 96 hpi.
